# The assessment of reliability generalisation of clinician-administered PTSD scale for DSM-5 (CAPS-5): a meta-analysis

**DOI:** 10.3389/fpsyg.2024.1354229

**Published:** 2024-08-09

**Authors:** Ajele Kenni Wojujutari, Erhabor Sunday Idemudia, Lawrence Ejike Ugwu

**Affiliations:** Faculty of Humanities, North-West University, Mafikeng, South Africa

**Keywords:** meta-analysis, reliability generalization, CAPS-5, Cronbach’s alpha coefficient, psychometric properties

## Abstract

**Background:**

The CAPS-5 is a reliable instrument for assessing PTSD symptoms, demonstrating strong consistency, validity, and reliability after a traumatic event. However, further research is warranted to explore the divergent validity of the CAPS-5 and its adaptation to diverse cultural contexts.

**Objective:**

In this meta-analysis, we endeavoured to comprehensively evaluate the reliability generalization of the CAPS-5 across diverse populations and clinical contexts.

**Methods:**

A reliability generalization meta-analysis on the psychometric properties of CAPS-5 was conducted, encompassing 15 studies. The original versions’ psychometric properties were systematically retrieved from databases including PubMed, PsychNet, Medline, CHAHL, ScienceDirect, Scopus, Web of Science, and Google Scholar, with a focus on studies published between 2013 and 2023. Two independent investigators evaluated study quality using QUADAS-2 and COSMIN RB, pre-registering the protocol in the Prospero database for transparency and minimizing bias risk.

**Results:**

Meta-analysis reveals CAPS-5 global reliability (α = 0.92, 95% CI [0.90, 0.94]), z = 99.44, *p* < 0.05 across 15 studies, supporting consistent internal consistency. Subscale analysis shows variability in Reexperiencing (α = 0.82), Avoidance (α = 0.68), Cognition and Mood (α = 0.82), and Hyperarousal (α = 0.74), with an overall estimate of 0.77 (95% CI [0.70;0.83]). Language-dependent analysis highlights reliability variations (α range: 0.83 to 0.92) across Brazilian-Portuguese, Dutch, English, French, German, Korean, and Portuguese. Test–retest reliability demonstrates stability (r = 0.82, 95% CI [0.79; 0.85]), with overall convergent validity (r = 0.59, 95% CI [0.50;0.68]).

**Conclusion:**

The meta-analysis affirms CAPS-5’s robust global and subscale reliability across studies and languages, with stable test–retest results. Moderator analysis finds no significant impact, yet substantial residual heterogeneity remains unexplained. Our findings contribute intricate insights into the psychometric properties of this instrument, offering a more complete understanding of its utility in PTSD assessment.

**Systematic review registration:**

https://www.crd.york.ac.uk/prospero/display_record.php?ID=CRD42023483748.

## Highlights

- This meta-analysis validates the high reliability of the Clinician-Administered PTSD Scale for DSM-5 for assessing PTSD symptoms, demonstrating a robust global reliability across different languages and clinical settings.
- The research provides detailed insights into the variability of subscale reliability for different PTSD symptoms, highlighting areas for focused clinical attention.
- The study confirms the CAPS-5’s adaptability and consistent performance across various cultural contexts, enhancing its utility for global clinical applications.

## 1 Introduction

The Clinician-Administered PTSD Scale for DSM-5 (CAPS-5) is a structured interview meticulously designed to assess the frequency and severity of each symptom of post-traumatic stress disorder (PTSD) within a one-month period following a traumatic event. Based on the criteria from the fifth edition of the Diagnostic and Statistical Manual of Mental Disorders (DSM-5), CAPS-5 has been validated across diverse populations, including trauma-exposed chronic pain patients, demonstrating robust psychometric properties such as inter-item consistency, convergent validity with self-report measures, and excellent test–retest reliability (Rivest-Beauregard et al., 2022; Cwik et al., 2023).

Despite its strengths, CAPS-5 is noted to have discrepancies in scoring compared to the PTSD Checklist for DSM-5 (PCL-5), with PCL-5 often reporting higher scores. These discrepancies are thought to arise from variations in item responses, scale anchors, and item wording, which can impact the interpretation and measurement of PTSD symptoms (Resick et al., 2023a,b). Discrepancies between these tools primarily stem from their methodological foundations the clinician-administered versus self-reported formats. The CAPS-5’s clinician-led approach may reduce bias and provide a more nuanced understanding of the patient’s condition, potentially leading to more accurate diagnoses (Weathers et al., 2018). In contrast, the self-administered nature of the PCL-5 may introduce bias, such as underreporting or overreporting symptoms, influenced by the patient’s self-awareness and stigma associated with PTSD (Kramer et al., 2022; Lee et al., 2022; Forkus et al., 2023).

Understanding and addressing these discrepancies is crucial for several reasons. Clinically, it informs the selection of the appropriate tool based on the context and specific needs CAPS-5 is preferable for in-depth assessments in therapeutic settings, whereas PCL-5 is suitable for initial evaluations and epidemiological studies where broad applicability is necessary (Caldas et al., 2020; Spies et al., 2020; Lee et al., 2022). From a research perspective, acknowledging these differences is essential for interpreting study outcomes, particularly in comparative analyses where tools may yield different results due to their inherent biases (Weathers et al., 2018; Caldas et al., 2020; Lee et al., 2022).

Building on the success of its predecessor, the CAPS-IV, CAPS-5 is deemed a psychometrically sound instrument for assessing PTSD severity in clinical settings. It has demonstrated consistent internal consistency, test–retest reliability, inter-rater reliability, and diagnostic accuracy across varied populations (Cwik et al., 2023; Hansen et al., 2023; Resick et al., 2023a,b). Nonetheless, ongoing challenges include aligning clinician-rated measures like the CAPS-5 with self-report measures, with issues stemming from differences in time-frame reminders, symptom comprehension, and trauma-related attribution errors (Kramer et al., 2022).

CAPS-5 uses specific dimensions or domains to diagnose PTSD according to DSM-5 criteria, with its subscales effectively measuring symptoms related to reexperiencing, avoidance, cognition and mood, and hyperarousal. These subscales have been validated for their high reliability, including test–retest and inter-rater reliability, in numerous studies (Hunt et al., 2018; Müller-Engelmann et al., 2018; Weathers et al., 2018; Kim et al., 2019; Spies et al., 2020; Zaman et al., 2020; Oliveira-Watanabe et al., 2021; Rivest-Beauregard et al., 2022; Krüger-Gottschalk, 2022; Lu et al., 2022).

Comparative studies reveal that CAPS-5 often scores higher than PCL-5, yet exhibits good convergent validity with it, suggesting that both tools assess similar constructs and provide comparable estimates of symptom change over time (Lee et al., 2022; Resick et al., 2023a,b). This similarity supports the use of CAPS-5 subscales for measuring PTSD symptoms effectively, as evidenced in various settings including among populations exposed to intimate partner violence (Ramírez et al., 2020; Martínez-Levy et al., 2021).

This current meta-analysis seeks to extend the existing literature by providing an in-depth examination of the reliability generalization of the CAPS-5, exploring potential sources of score discrepancies, and synthesizing findings across diverse populations and clinical contexts. The CAPS-5 has shown excellent psychometric properties not only in English but also in its French and German versions, as well as the European Portuguese version designed for diagnosing PTSD in children and adolescents, indicating its broad applicability and robustness across different cultural contexts (McNally, 2014; Müller-Engelmann et al., 2018; Barroca A. et al., 2022; Rivest-Beauregard et al., 2022; Cwik et al., 2023).

Through this meta-analysis, we aim to provide valuable insights into the reliability generalization of CAPS-5, enhancing understanding of its performance across diverse settings, and supporting its ongoing adaptation and use in global mental health research.

## 2 Methods

We conducted a reliability generalization meta-analysis (RG) to assess the psychometric properties of the Clinician-Administered PTSD Scale for DSM-5 (CAPS-5). This meta-analysis included data from 15 studies, and the review method adhered to reliability COnsensus-based Standards for the selection of Health Measurement Instruments Risk of Bias checklist (COSMIN RB, Mokkink et al., 2018).

The study protocol, including the specific methods for the reliability generalization meta-analysis, was pre-registered in the Prospero database (registration number CRD42023483748). This pre-registration ensures transparency and minimizes the risk of bias in the study design and analysis.

### 2.1 Search strategy

To achieve a comprehensive coverage of the literature, a thorough exploration was conducted across various databases. The databases referenced are PubMed, PsychNet, Medline, CHAHL, ScienceDirect, Scopus, Web of Science, and Google Scholar. The systematic review was meticulously designed with a predetermined search strategy focused on evaluating the reliability of the Clinician-Administered PTSD Scale for DSM-5 (CAPS-5) across varied populations. The aim was to encompass a wide range of studies that scrutinized the psychometric properties, particularly the reliability, of CAPS-5. To accomplish this, an extensive exploration of numerous prominent databases was executed from January 2013 to December 2023. The commencement year was chosen to align with subsequent research on DSM-5 implementation, ensuring that the data reflected the most up-to-date diagnostic criteria. The exploration strategy encompassed a blend of pivotal terms and expressions to encompass all pertinent studies. The key terms and search expressions comprised: “Clinician-Administered PTSD Scale for DSM-5 CAPS-5,” “CAPS-5,” “reliability,” “psychometrics properties,” “Validity,” “internal consistency,” and “test–retest reliability” were employed. Moreover, the amalgamation of keywords with Boolean operators in the search strategy, such as (“Clinician-Administered PTSD Scale for DSM-5 CAPS-5,” OR “CAPS-5”) AND (“reliability” OR “internal consistency” OR “test–retest reliability” OR “psychometrics properties”) were used.

### 2.2 Selection criteria

The study aims to include studies on the psychometric properties and reliability assessment of the Clinician-Administered PTSD Scale for DSM-5 (CAPS-5) in any population or setting, including validation, test–retest reliability, internal consistency, and cross-cultural validation studies. Exclusion criteria include studies that do not report on reliability or psychometric properties, as well as publications not in English, reviews, conference abstracts, editorials, case reports, and those with insufficient data. The inclusion of only English papers is primarily due to the accessibility and common usage of these studies in international research. Translating and adapting the CAPS-5 into different languages involves rigorous validation processes that were beyond the scope of this review.

### 2.3 Data extraction

The selection studies for RG were based on eligibility criteria, with two independent reviewers screening titles and abstracts. Full-text articles were assessed for final inclusion, with discrepancies resolved through consultation with a third reviewer. Data extraction focuses on the psychometric properties of the CAPS-5, capturing details such as study characteristics, demographic information, reliability coefficients, and validity measures. Analysis will distinguish between clinical and non-clinical samples to assess differences in psychometric properties. The process was documented for consistency and accuracy, and disagreements were resolved by consensus between reviewers.

### 2.4 Quality assessment

Two independent investigators conducted a rigorous evaluation of study quality using the Quality Assessment of Diagnostic Accuracy Studies (QUADAS-2, Whiting et al., 2021) and the COnsensus-based Standards for the selection of Health Measurement Instruments Risk of Bias checklist (COSMIN RB, Mokkink et al., 2018). This critical examination aimed to provide an in-depth analysis of the methodological robustness and potential biases in the included studies.

**Figure 1:**
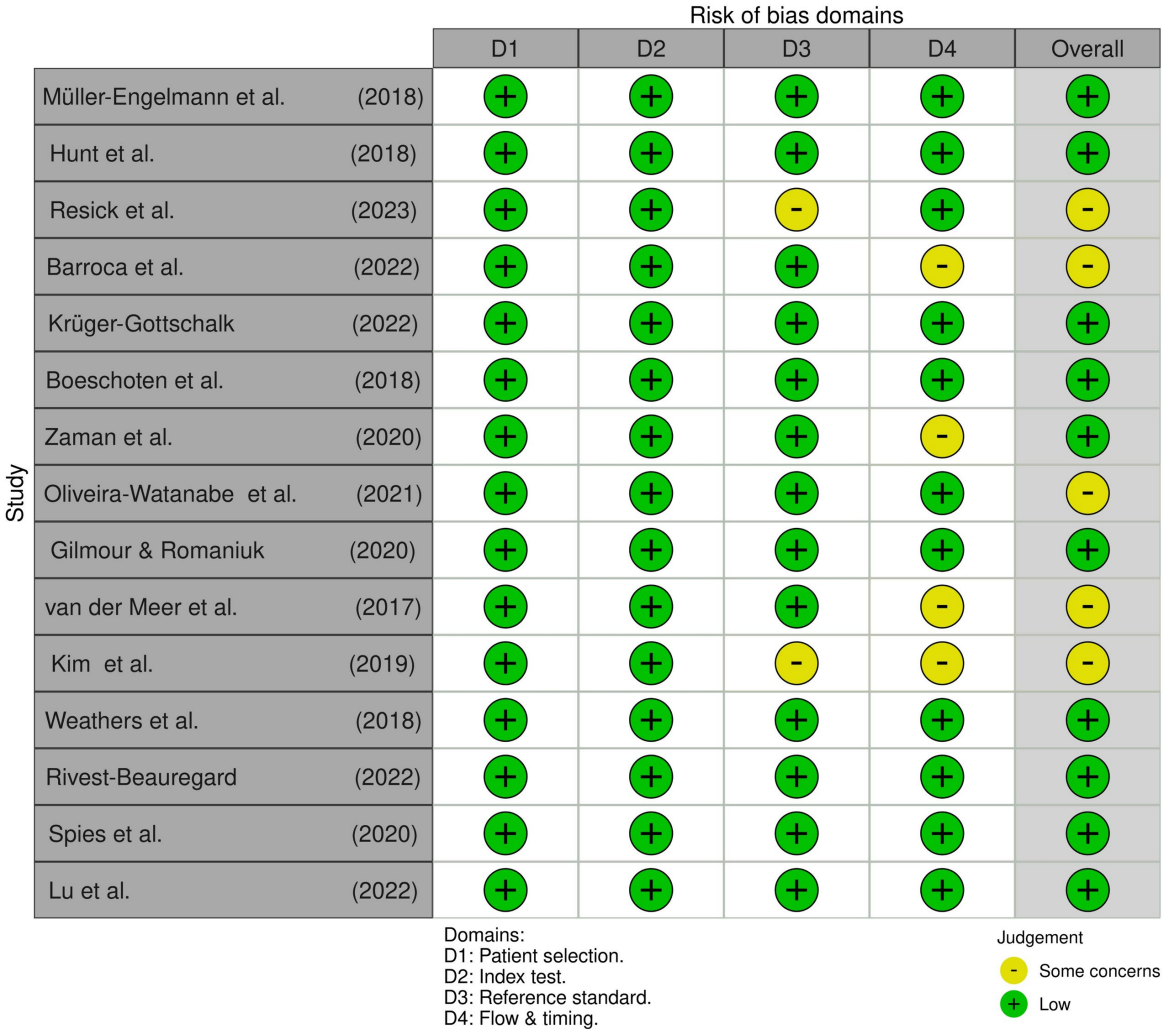
QUADAS-2 assessments for included studies.

Figure 1 meticulously illustrates the comprehensive assessment of multiple studies through the QUADAS-2 framework. This tool, specifically designed for diagnostic accuracy studies, scrutinizes patients’ selection, Index Test, Reference Standard, Flow and Timing, and Overall Assessment. Utilizing a 4-point scale (“Low,” “Some concern,” and “High”), each study underwent a thorough evaluation, revealing critical insights into methodological rigour and inherent biases.

Despite the apparent methodological scrutiny, it is crucial to note that all studies received a classification of “Low,” signalling a favourable outcome for methodological quality. While this may suggest a positive assessment, it is imperative to interpret this result with caution, considering the potential implications for research integrity and the reliability of the findings (Whiting et al., 2021).

Turning attention to the COSMIN RB checklist, the focus was on measurement properties, including reliability, validity, and responsiveness. The checklist’s 10 checkboxes delve into various design and statistical method aspects, providing a nuanced evaluation.

Table 1 presents systematic ratings for reliability, validity, and responsiveness, classifying each study as “Very Good,” “Adequate,” or “Doubtful.” These classifications offer a critical perspective on the methodological strengths and weaknesses of the studies, shedding light on potential limitations in the reliability, validity, and responsiveness of the measurement properties under investigation.

**Table 1:** Summary of COSMIN risk of bias (RB) checklist assessments for included studies.

Author (Year)	Sample size	Reliability	Validity	Responsiveness
Müller-Engelmann et al. (2018)	274	Very Good	Very Good	Very good
Hunt et al. (2018)	309	Very Good	Very good	Very good
Resick et al. (2023a, 2023b)	739	Very Good	Very good	Very good
Barroca A. et al. (2022)	101	Adequate	Adequate	Adequate
Krüger-Gottschalk (2022)	345	Very Good	Very good	Very good
Boeschoten et al. (2018)	669	Very Good	Adequate	Adequate
Zaman et al. (2020)	140	Adequate	Very good	Very good
Oliveira-Watanabe et al. (2021)	128	Very Good	Very good	Very good
Gilmour and Romaniuk (2020)	267	Very Good	Adequate	Adequate
van der Meer et al. (2017)	89	Adequate	Adequate	Adequate
Kim et al. (2019)	274	Very Good	Very good	Very good
Weathers et al. (2018)	867	Very Good	Very good	Very good
Rivest-Beauregard et al. (2022)	168	Very Good	Very good	Very good
Spies et al. (2020)	219	Very Good	Very good	Very good
Lu et al. (2022)	536	Very Good	Very good	Very good

Considering these critical evaluations, it is imperative for researchers to consider the implications for the reliability and credibility of the included studies. While the assessments may indicate a certain level of methodological rigour, deeper scrutiny is warranted to ensure the robustness of the findings and their applicability in the broader context of diagnostic accuracy of health measurement instruments.

### 2.5 PICOS framework

Table 2 provides a comprehensive overview of the PICOS framework for a meta-analysis on the reliability generalization of the Clinician-Administered PTSD Scale for DSM-5 (CAPS-5). It includes individuals with PTSD from varied demographics such as military veterans and trauma survivors, assessing the use of CAPS-5 in clinical and research settings for effectiveness and reliability. The analysis compares CAPS-5 against other PTSD assessment tools like the PTSD Checklist for DSM-5 (PCL-5), focusing on differences in reliability, validity, and diagnostic outcomes. The outcomes examined are CAPS-5’s psychometric properties, including internal consistency, test–retest reliability, inter-rater reliability, construct, criterion, content validity, sensitivity, specificity, and predictive values. The study designs involve observational studies using CAPS-5, including cross-sectional, cohort, and case–control studies, as well as comparative assessments of CAPS-5 against other diagnostic tools. This framework supports an in-depth evaluation of CAPS-5’s utility and accuracy in diagnosing PTSD across diverse populations and study designs.

**Table 2:** PICOS framework for meta-analysis on the reliability generalization of CAPS-5.

Component	Description
Population	Individuals diagnosed with PTSD across diverse demographics including military veterans, survivors of trauma such as abuse, accidents, and disasters, and clinical populations from varied cultural backgrounds. The study encompasses various age groups and both genders.
Intervention	Use of the Clinician-Administered PTSD Scale for DSM-5 (CAPS-5) as a diagnostic tool in clinical and research settings to assess its effectiveness and reliability in diagnosing PTSD according to DSM-5 criteria.
Comparators	Other psychometric properties derived from different PTSD assessment tools, specifically comparing CAPS-5 with self-report measures like the PTSD Checklist for DSM-5 (PCL-5), focusing on differences in reliability, validity, and diagnostic outcomes.
Outcomes	Psychometric properties of CAPS-5, specifically focusing on: Reliability: Internal consistency, test–retest reliability, inter-rater reliability Validity: Construct, criterion, and content validity Diagnostic Accuracy: Sensitivity, specificity, and predictive values across various populations.
Study designs	Observational studies that have employed CAPS-5 in assessing PTSD, including cross-sectional, cohort, and case–control studies. Studies performing comparative assessments of CAPS-5 against other diagnostic tools are also included.

### 2.6 Data analysis

We applied statistical methods for meta-analysis to synthesize the findings from multiple studies. Effect sizes were calculated for validity and reliability coefficients, with random-effects models and Mixed-Effects Model being used to account for heterogeneity between studies. A qualitative analysis will be used to summarize and interpret the reliability of the Clinician-Administered PTSD Scale for DSM-5 (CAPS-5). Also, Meta-analysis will be considered, using a random-effects model to pool reliability coefficients. Heterogeneity will be explored using the I^2^ statistic and subgroup analyses. Statistical software R studio metafor-package was used for the data analysis.

## 3 Results

### 3.1 Reliability generalization meta-analysis: selection and induction

**Figure 2:**
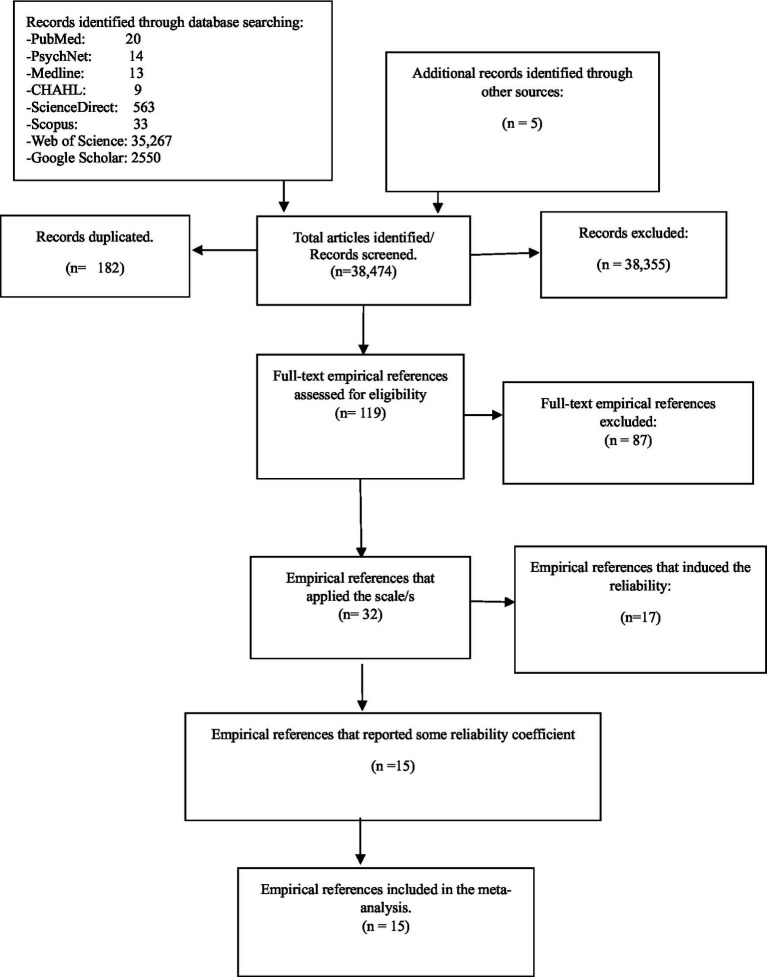
REGEMA flowchart of the studies selection process.

This meta-analysis on reliability generalization (RG) encompassed 15 studies that reported reliability coefficients, obtained from various databases (Figure 2). Among these studies, five focused on the CAPS-5 with 30 items, yielding Cronbach’s alpha values ranging from 0.89 to 0.90 (van der Meer et al., 2017; Boeschoten et al., 2018; Gilmour and Romaniuk, 2020; Barroca I. et al., 2022). The remaining 10 studies were centred on CAPS-5 with 20 items, reporting Cronbach’s alpha values ranging from 0.83 to 0.97 (Hunt et al., 2018; Müller-Engelmann et al., 2018; Weathers et al., 2018; Kim et al., 2019; Spies et al., 2020; Zaman et al., 2020; Oliveira-Watanabe et al., 2021; Rivest-Beauregard et al., 2022; Resick et al., 2023a,b; Krüger-Gottschalk, 2022; Lu et al., 2022). The five studies reporting reliability coefficients for the subscales of CAPS-5 were conducted using the 20-items version. Additionally, the five studies reporting test–retest reliability for both the total CAPS-5 scores and subscales were conducted using the 20-items version. Mean age and gender (women) information of participants were reported in only 12 of the included studies.

Table 3 demonstrates the countries and populations included in the research utilizing the Clinician-Administered PTSD Scale for DSM-5 (CAPS-5). These studies span a variety of countries – Germany, USA, Portugal, Netherlands, Pakistan, Brazil, Australia, South Korea, France, Lebanon, and Canada. This indicates a comprehensive geographical validation of CAPS-5. The populations studied include trauma-exposed individuals, military veterans, children and adolescents, trauma survivors, individuals recently exposed to traumatic events, police officers, and individuals seeking PTSD assessment and treatment, demonstrating the wide applicability of CAPS-5 across different demographic and clinical groups. All studies focus on validating CAPS-5, assessing its internal consistency and test–retest reliability, and some also focus on cross-cultural validation, which is crucial for its use in diverse settings. Studies consistently report good internal consistency for CAPS-5, confirming its reliability, and high test–retest reliability, indicating stability over time. Several studies validate CAPS-5 in non-English-speaking contexts, ensuring its effectiveness across different languages and cultures. This comprehensive validation and reliability assessment supports the use of CAPS-5 as a robust tool for diagnosing PTSD across diverse populations and settings.

**Table 3:** Countries and populations of included studies.

S/N	Author (Year)	Country	Population
1	Müller-Engelmann et al. (2018)	Germany	Trauma-exposed sample
2	Hunt et al. (2018)	USA	Adult injured trauma survivors
3	Resick et al. (2023a,b)	USA	Military and veteran treatment-seeking samples
4	Barroca I. et al. (2022)	Portugal	Children and adolescents
5	Krüger-Gottschalk et al. (2017)	Germany	Diverse trauma-exposed individuals
6	Boeschoten et al. (2018)	Netherlands	Trauma-exposed individuals
7	Zaman et al. (2020)	Pakistan	Trauma survivors (life-threatening trauma)
8	Oliveira-Watanabe et al. (2021)	Brazil	Individuals recently exposed to traumatic events
9	Gilmour and Romaniuk (2020)	Australia	Australian Vietnam veterans
10	van der Meer et al. (2017)	Netherlands	Referred police officers
11	Kim et al. (2019)	South Korea	PTSD, mood disorder, anxiety disorder, and healthy controls
12	Weathers et al. (2018)	USA	Military veterans
13	Rivest-Beauregard et al. (2022)	France, Lebanon, Canada	Individuals seeking PTSD assessment and/or treatment

### 3.2 Overall reliability for CAPS-5

**Figure 3:**
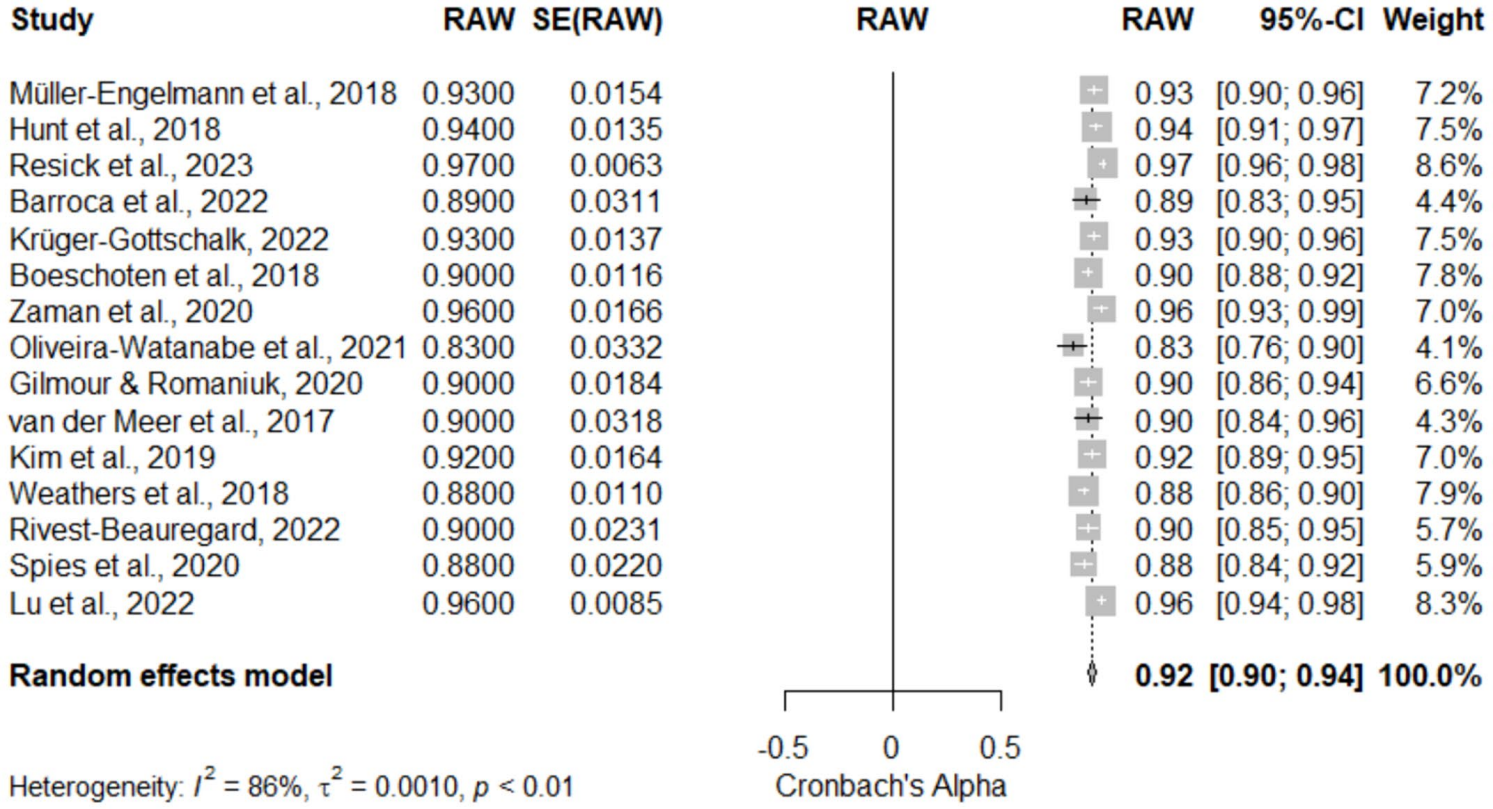
Forest plot for meta-analysis of the reliability of the CAPS-5 across studies included.

**Figure 4:**
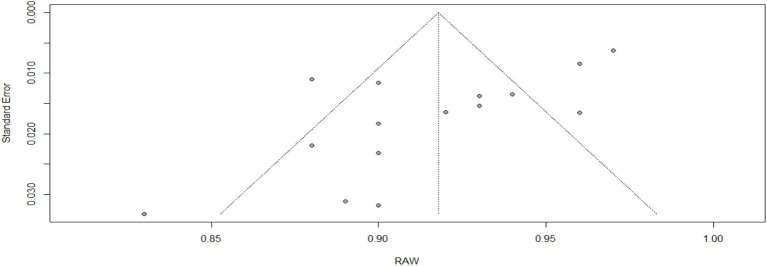
Funnel plot for meta-analysis of the reliability of the CAPS-5 across studies included.

Table 4, Figure 3 (Forest Plot), and Figure 4 (Funnel Plot): Meta-Analysis of the Reliability of the Clinician-Administered PTSD Scale for DSM-5 (CAPS-5). Table 4 and Figure 3 demonstrate the reliability of the CAPS-5 across 15 studies, showcasing a high pooled reliability coefficient of 0.92 (95% CI [0.90, 0.94]), z = 99.44, *p* < 0.05, indicative of the CAPS-5’s robust performance in diverse clinical settings. The forest plot (Figure 3) visualizes individual study effects along with the pooled result, confirming consistently high reliability across studies. Significant heterogeneity was noted (I^2^ = 86.4%, τ^2^ = 0.0010), highlighting variability among studies that might be due to differences in study designs or populations. The funnel plot (Figure 4) assesses publication bias, showing a symmetric distribution of studies around the pooled estimate, suggesting minimal bias. This meta-analysis underscores the CAPS-5’s effective use in varied demographic and clinical contexts, supporting its broad applicability in PTSD assessment.

**Table 4:** Mean reliability and heterogeneity across included studies.

Total scale/Subscales	k	Estimate (α_+_)	z values	90%CL	Q	I^2^	τ^2^
				LL (UL)			
**Coefficient alpha**							
CAPS-5 (Global)	15	0.92	99.44**	[0.90; 0.94]	102.69	86.4%	0.0010
CAPS-5 (30-Items)	5	0.90		[0.89; 0.92]			
CAPS-5 (20-Items)	10	0.91		[0.91; 0.92]			
Model	2	0.91	156.32**	[0.90;0.92]	102.69	53.6%	0.0001
B = Cluster	10	0.82		[0.70;0.83]			
C = Cluster	10	0.68		[0.66;0.70]			
D = Cluster	10	0.82		[0.81;0.83]			
E = Cluster	10	0.74		[0.72;0.76]			
Model	4	0.77	22.50**	[0.70;0.83]	213.34**	98.6%	0.0046
**Test–Retest**							
B = Cluster	2	0.85		[0.84;0.88]			
C = Cluster	2	0.78		[0.75;0.80]			
D = Cluster	2	0.83		[0.81;0.85]			
E = Cluster		0.78		[0.76;0.81]			
CAPS-5 Total	5	0.85		[0.83;0.87]			
Model	5	0.82	49.85**	[0.79;0.86]	35.40**	88.7%	0.0012

**p <0.001.

### 3.3 Meta-analysis of reliability comparison between CAPS-5-30 and CAPS-5-20

**Figure 5:**
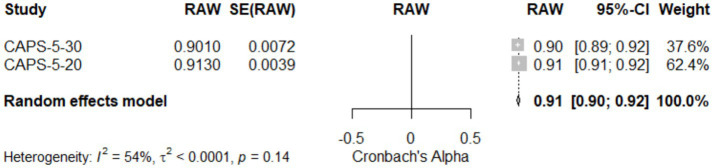
Forest plot for meta-Analysis of the reliability between CAPS-5-30 and CAPS-5-20.

Table 4 and Figure 5: Meta-Analysis of reliability comparison between CAPS-5-30 and CAPS-5-20. This meta-analysis assessed the reliability of two versions of the Clinician-Administered PTSD Scale for DSM-5 (CAPS-5), with 30 items (CAPS-5-30) and 20 items (CAPS-5-20). The results, illustrated in Table 4 and visualized in Figure 5 (Forest Plot), indicate that CAPS-5-20 exhibited slightly higher reliability (0.91, 95% CI [0.91; 0.92]) compared to CAPS-5-30 (0.90, 95% CI [0.89; 0.92]). The random effects model, reflecting pooled data from two studies, showed an overall reliability coefficient of 0.92 (95% CI [0.90; 0.92]), with a z-score of 156.32, indicating statistically significant high reliability. The analysis revealed moderate heterogeneity (I^2^ = 53.6%; τ^2^ = 0.0001), suggesting some variability between the studies, which may be attributed to the difference in the number of items between the two CAPS versions. The test of heterogeneity (Q = 2.16, df = 1, *p* = 0.14) did not show significant differences, supporting the robustness of the findings. These results underscore the reliability of both CAPS-5 versions in clinical settings, with CAPS-5-20 showing marginally higher consistency.

### 3.4 Meta-analysis of subscale-specific reliability

**Figure 6:**
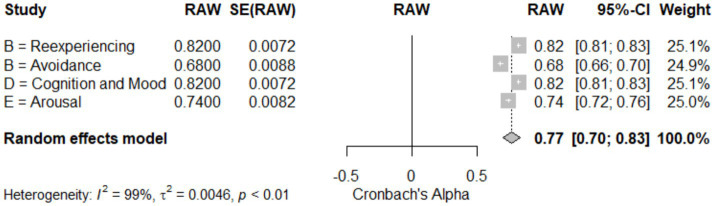
Forest plot for meta-analysis of the subscales reliability (Cronbach’s Alpha).

Table 4 and Figure 6: Meta-Analysis of Subscale-Specific Reliability for CAPS-5. This meta-analysis evaluated the reliability of different subscales of the CAPS-5, assessing symptoms of PTSD in various domains. The reexperiencing (Cluster B) and cognition and mood (Cluster D) subscales both demonstrated high reliability, with coefficients of 0.82 (95% CI [0.81; 0.83]). By contrast, the avoidance (Cluster B) subscale showed lower reliability at 0.68 (95% CI [0.66; 0.70]), and the arousal (Cluster E) subscale had a reliability of 0.74 (95% CI [0.72; 0.76]) (see Table 4). The pooled data from four studies (k = 4) indicated an overall reliability of 0.77 (95% CI [0.70; 0.83]), with a z-score of 22.50, *p* < 0.05, suggesting significant reliability across subscales. The analysis displayed a very high heterogeneity (I^2^ = 98.6%; τ^2^ = 0.0046), which is visualized in Figure 6 (Forest Plot), reflecting considerable variability among the studies. This high heterogeneity might be attributed to differences in study populations or assessment procedures, highlighting the need for nuanced interpretation of each subscale’s reliability in diverse clinical settings.

### 3.5 Test–retest reliability

**Figure 7:**
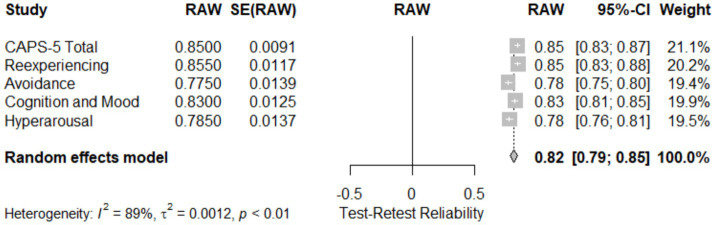
Forest plot for meta-analysis of the subscales reliability (Test-Retest Reliability).

Table 4 and Figure 7: Meta-Analysis of subscale-specific test–retest reliability for CAPS-5. This meta-analysis assessed the test–retest reliability of the CAPS-5 total score and its subscales across five studies. The total CAPS-5 score showed high reliability (0.850; 95% CI [0.83; 0.87]). Subscale reliability varied, with the reexperiencing subscale showing slightly higher reliability (0.86; 95% CI [0.83; 0.88]) compared to cognition and mood (0.830; 95% CI [0.81; 0.85]) and hyperarousal (0.79; 95% CI [0.76; 0.81]). The avoidance subscale demonstrated the lowest reliability (0.76; 95% CI [0.75; 0.80]). The pooled effect size across all subscales and studies was 0.82 (95% CI [0.79; 0.85]), with a z-score of 49.85, *p* < 0.05, indicating substantial reliability. Heterogeneity was high (I^2^ = 88.7%), as quantified by τ^2^ = 0.0012 and H = 2.97, suggesting significant variation across studies, likely reflecting differences in assessment intervals or sample characteristics. Figure 7 (Forest Plot) visually presents these findings, illustrating the distribution of effect sizes and the consistency of test–retest reliability across the studies.

### 3.6 Language-dependent analysis of CAPS-5 reliability

**Figure 8:**
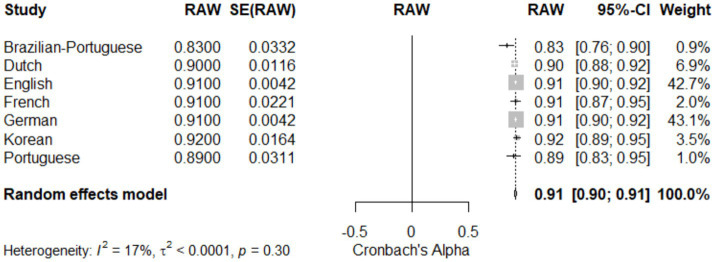
Forest plot for meta-analysis of the CAPS-5 reliability across language versions.

Table 5 and Figure 8: Language-Dependent Analysis of CAPS-5 Reliability. This meta-analysis evaluates the reliability of the CAPS-5 across seven different language versions. The random effects model showed a pooled reliability estimate of 0.91 (95% CI [0.90; 0.91]), indicating high consistency across languages with a z-score of 293.11, *p* < 0.05. Individual language reliabilities were as follows: Brazilian-Portuguese (0.83; 95% CI [0.76; 0.90]), Dutch (0.90; 95% CI [0.88; 0.92]), English (0.91; 95% CI [0.90; 0.92]), French (0.91; 95% CI [0.87; 0.95]), German (0.91; 95% CI [0.90; 0.92]), Korean (0.92; 95% CI [0.89; 0.95]), and Portuguese (0.89; 95% CI [0.83; 0.95]). Heterogeneity across studies was low (I^2^ = 16.7%), as quantified by τ^2^ = 0.0001 and H = 1.10, suggesting minimal variability in reliability between the translations. Figure 8 (Forest Plot) visually presents these findings, emphasizing the robust reliability of CAPS-5 across diverse linguistic contexts and contributing to its validity as a global diagnostic tool for PTSD.

**Table 5:** Mean reliability and heterogeneity across language versions.

Language Version	k	Estimate (α_+_)	z values	90%CL	Q	I^2^	τ^2^
				LL (UL)			
Model	7	0.91	293.11**	[0.90;0.91]	7.20	16.7%	0.0001
Brazilian-Portuguese	1	0.83		[0.76;0.90]			
Dutch	1	0.90		[0.88;0.92]			
English	4	0.91		[0.90;0.92]			
French	1	0.91		[0.87;0.95]			
German	3	0.91		[0.90;0.92]			
Korean	1	0.92		[0.89;0.95]			
Portuguese	1	0.89		[0.83;0.95]			

**p <0.001.

### 3.7 Meta-analysis of convergent validity of CAPS-5

**Figure 9:**
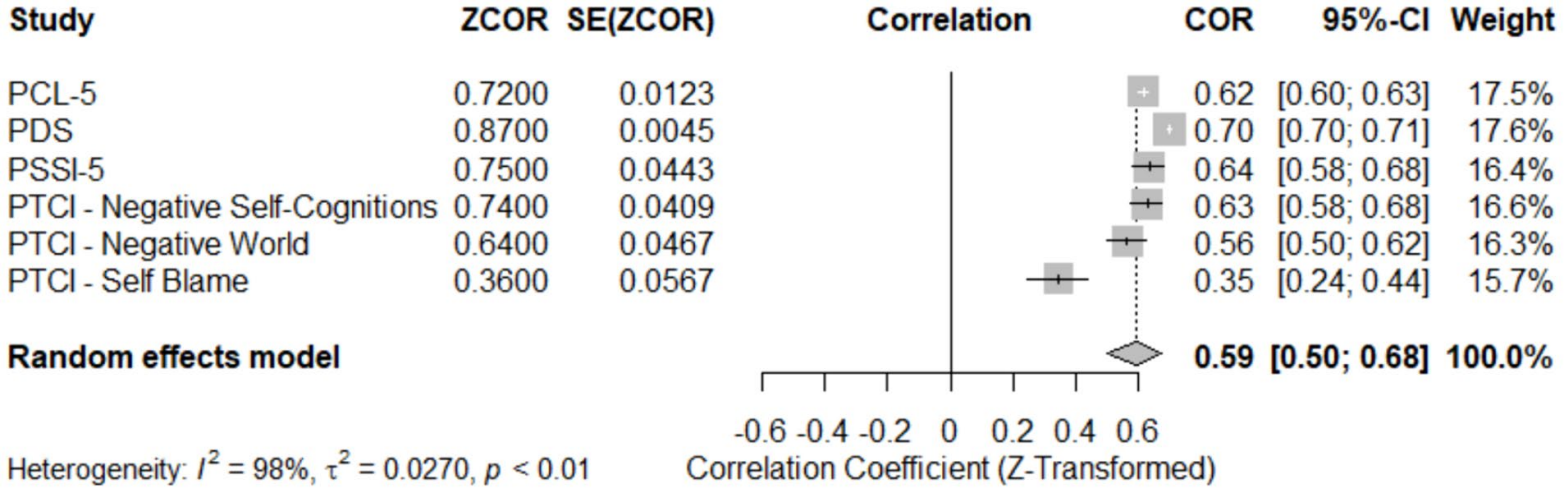
Forest plot for meta-analysis of convergent validity of CAPS-5.

Table 6 and Figure 9: Meta-Analysis of Convergent Validity of CAPS-5. This analysis assessed the convergent validity of the CAPS-5 by comparing it with several well-established PTSD measures. The random effects model estimated a pooled correlation coefficient of 0.59 (95% CI [0.50; 0.68]), z = 9.94, *p* < 0.05, indicating moderate to strong convergent validity across the measures. The measures analysed included the PTSD Checklist for DSM-5 (PCL-5) with a correlation of 0.62 (95% CI [0.60; 0.63]), the Posttraumatic Diagnostic Scale (PDS) at 0.70 (95% CI [0.70; 0.71]), and the PTSD Symptom Scale Interview for DSM-5 (PSSI-5) with 0.6351 (95% CI [0.58; 0.68]). Also analysed were subscales of the Posttraumatic Cognitions Inventory (PTCI), showing varied correlations: Negative Self-Cognitions at 0.63 (95% CI [0.58; 0.68]), Negative World at 0.56 (95% CI [0.50; 0.62]), and Self Blame at 0.35 (95% CI [0.24; 0.44]). The forest plot in Figure 9 visually represents each measure’s contribution and the variability among the correlations. Significant heterogeneity (I^2^ = 97.9%, Q = 237.59, *p* < 0.05) suggests substantial differences in how these measures correlate with the CAPS-5, underscoring the need for careful consideration of the specific PTSD symptoms and cognitions assessed by different tools.

**Table 6:** Mean reliability and heterogeneity across language versions.

Language version	k	Estimate (r_+_)	z values	90%CL	Q	I^2^	τ^2^
				LL (UL)			
Model	6	0.59	9.94 **	[0.50;0.68]	9.94**	97.9%	0.0270
PCL-5	8	0.62		[0.60;0.63]			
PDS	1	0.70		[0.70;0.71]			
PSSI-5	2	0.64		[0.58;0.68]			
PTCI-Negative Self-Cognitions	1	0.63		[0.58;0.68]			
PTCI-Negative World	1	0.56		[0.50;0.62]			
PTCI-Self Blame	1	0.35		[0.24;0.44]			

**p <0.001.

## 4 Discussion

The present meta-analysis sought to conduct a thorough evaluation of the reliability and validity of the Clinician-Administered PTSD Scale for DSM-5 (CAPS-5) across diverse populations and clinical contexts. The findings offer substantial insights into the instrument’s efficacy and contribute to the broader landscape of PTSD assessment.

Consistent with previous validation studies, the CAPS-5 demonstrated a high overall reliability, evidenced by a global Cronbach’s alpha estimate of 0.91 across 15 studies. This aligns with a body of research highlighting the instrument’s robust psychometric properties, encompassing internal consistency, test–retest reliability, and inter-rater reliability (Rivest-Beauregard et al., 2022; Cwik et al., 2023; Resick et al., 2023a,b). Notably, the meta-regression analysis revealed that neither mean age nor gender significantly moderated Cronbach’s alpha, emphasizing the consistency of the CAPS-5’s reliability across different demographic groups. Despite the observed score discrepancies between the CAPS-5 and the PTSD Checklist for DSM-5 (PCL-5), with the former producing higher scores, the CAPS-5 remains a reliable diagnostic tool for assessing PTSD severity. The robust overall reliability estimates attest to the instrument’s continued utility in clinical settings.

The adoption of a cluster-based approach for subscale analysis provided a nuanced examination of the CAPS-5’s internal consistency. The subscales, including Reexperiencing (B), Avoidance (C), Cognition and Mood (D), and Hyperarousal (E), displayed varying reliability estimates ranging from 0.68 to 0.82. This variability suggests a moderate to high level of internal consistency across specific symptom clusters. This variability suggests a moderate to high level of internal consistency across distinct symptom clusters. This finding resonates with prior research affirming the CAPS-5’s robust psychometric properties, including subscale reliability, test–retest reliability, and inter-rater reliability (Hunt et al., 2018; Müller-Engelmann et al., 2018; Weathers et al., 2018; Kim et al., 2019; Spies et al., 2020; Zaman et al., 2020; Oliveira-Watanabe et al., 2021; Rivest-Beauregard et al., 2022; Resick et al., 2023a,b; Krüger-Gottschalk, 2022; Lu et al., 2022). However, the substantial total heterogeneity observed underscores the need for further investigation into factors contributing to variations in subscale reliability, such as population differences, trauma types, or cultural contexts. Understanding the reliability of subscales is imperative for clinicians to accurately assess specific PTSD symptom domains. The identified heterogeneity calls for additional research to delve into the intricacies of factors influencing variations in subscale reliability across different studies.

The evaluation of the CAPS-5 across different languages and cultural contexts reinforces its adaptability and psychometric soundness. Our analysis revealed positive psychometric properties in the French and German versions, supporting the cross-cultural utility of the instrument (Barroca A. et al., 2022; Cwik et al., 2023). Additionally, the European Portuguese version exhibited commendable internal consistency and suitability for diagnosing PTSD in children and adolescents (McNally, 2014). The CAPS-5’s efficacy in assessing post-traumatic symptomatology in women exposed to intimate partner violence further bolsters its cross-cultural validity (Ramírez et al., 2020).

The meta-analysis underscores the CAPS-5’s substantial convergent validity compared with established PTSD measures yet reveals significant heterogeneity (I^2^ = 97.9%), suggesting variability in its performance across different contexts and populations (Lee et al., 2022; Resick et al., 2023a,b). The varied correlations, particularly in the PTCI subscales such as Self Blame, highlight potential limitations in CAPS-5’s ability to capture specific cognitive dimensions of PTSD. This discrepancy may reflect divergent constructs assessed by these instruments, indicating that CAPS-5 might not fully capture certain cognitive aspects of PTSD, which are crucial for treatment outcomes (Kramer et al., 2022; Forkus et al., 2023).

The relatively lower correlation for the Self Blame subscale suggests a gap in CAPS-5’s assessment of self-directed blame, a key component of post-traumatic cognition. This finding suggests that clinicians and researchers might need to supplement CAPS-5 with additional measures for a more comprehensive evaluation of PTSD cognitions, especially when such aspects are clinically significant (Ramírez et al., 2020; Martínez-Levy et al., 2021).

Despite CAPS-5’s robust utility across various languages and cultural contexts, the moderate convergent validity reported calls for a critical review of cultural influences on the interpretation of PTSD symptoms and the effectiveness of standardized measures like CAPS-5 in diverse settings (McNally, 2014; Cwik et al., 2023). Cultural sensitivity in diagnostic tools is crucial, as evidenced by performance variations observed in non-English versions of CAPS-5. These findings support the CAPS-5 as a valuable diagnostic tool but also highlight the necessity for cautious application and further research to enhance PTSD assessment tools across diverse clinical contexts and populations (Weathers et al., 2018; Caldas et al., 2020).

### 4.1 Limitations and future directions

The evaluation of the CAPS-5 in various linguistic and cultural contexts underscores its adaptability and robust psychometric properties, enhancing our understanding of PTSD assessment in diverse global settings. This adaptability is crucial for the advancement of PTSD intervention and care in diverse cultural contexts. Nevertheless, this systematic review encounters constraints that require careful consideration. The limited quantity of studies, predominantly conducted between 2013 and 2023, may limit the generalizability of the findings. This narrow timeframe may overlook earlier influential research, and fails to encompass the entire spectrum of existing literature, potentially distorting the perception of the effectiveness of CAPS-5.

Furthermore, the observed diversity in subscale consistency indicates variations in how distinct populations react to the diagnostic instrument, highlighting the necessity for further exploration of contextual and demographic factors influencing specific symptom groupings. This diversity emphasizes the importance of conducting more thorough investigations into these elements.

Future investigations should aim to include a more extensive range of studies that go beyond the strict recent publication requirements, analyzing how different cultural and linguistic modifications of the CAPS-5 impact its diagnostic precision and credibility. Broadening the research scope can furnish a more detailed insight into the instrument’s usefulness and bolster its implementation in clinical environments globally, ultimately resulting in more personalized and efficacious PTSD interventions.

## 5 Conclusion

The meta-analysis provides robust evidence supporting the overall reliability and validity of the Clinician-Administered PTSD Scale for DSM-5 (CAPS-5). The instrument demonstrates consistent reliability across diverse populations, with the subscale analysis offering nuanced insights into the internal consistency of specific symptom clusters. Despite observed discrepancies in scores with self-report measures and identified gaps in the literature, the CAPS-5 remains a reliable tool for diagnosing and assessing PTSD severity. The findings underscore the importance of continued research to refine and expand our understanding of PTSD assessment instruments in varied clinical and cultural contexts.

## Data availability statement

The original contributions presented in the study are included in the article/supplementary material, further inquiries can be directed to the corresponding author.

## Author contributions

AW: Conceptualization, Data curation, Formal analysis, Methodology, Software, Validation, Visualization, Writing – original draft, Writing – review & editing. EI: Conceptualization, Supervision, Writing – review & editing. LU: Conceptualization, Data curation, Methodology, Validation, Writing – review & editing.
